# Comparison of the Pathogenicity in Mice of A(H1N1)pdm09 Viruses Isolated between 2009 and 2015 in Japan

**DOI:** 10.3390/v12020155

**Published:** 2020-01-29

**Authors:** Hiromichi Mitake, Atsuhiro Yasuhara, Tiago J. S. Lopes, Yuko Tagawa-Sakai, Kohei Shimizu, Hiroki Ozawa, Chiharu Kawakami, Saeko Morikawa, Norio Sugaya, Tokiko Watanabe, Yoshihiro Kawaoka

**Affiliations:** 1 Division of Virology, Department of Microbiology and Immunology, Institute of Medical Science, University of Tokyo, Tokyo 108-8639, Japan; srdyh595@yahoo.co.jp (H.M.); yasuhara@ims.u-tokyo.ac.jp (A.Y.); ytsakai@ims.u-tokyo.ac.jp (Y.T.-S.); 2 Department of Pathobiological Sciences, School of Veterinary Medicine, University of Wisconsin-Madison, Madison, WI 53711, USA; tiagojose.dasilvalopes@wisc.edu; 3 Yokohama City Institute of Public Health, Kanagawa 236-0051, Japan; ko05-shimizu@city.yokohama.jp (K.S.); hi07-ozawa@city.yokohama.jp (H.O.); ch00-kawakami@city.yokohama.jp (C.K.); 4 Division of Virology, Department of Microbiology, Osaka Institute of Public Health, Osaka 537-0025, Japan; morikawa@iph.osaka.jp; 5 Department of Infection Control and Pediatrics, Keiyu Hospital, Yokohama 220-0012, Japan; sugaya-n@za2.so-net.ne.jp; 6 Department of Special Pathogens, International Research Center for Infectious Diseases, Institute of Medical Science, University of Tokyo, Tokyo 108-8639, Japan

**Keywords:** influenza, pandemic virus, pathogenicity

## Abstract

The A(H1N1)pdm09 virus emerged in 2009 and continues to circulate in human populations. Recent A(H1N1)pdm09 viruses, that is, A(H1N1)pdm09 viruses circulating in the post-pandemic era, can cause more or less severe infections than those caused by the initial pandemic viruses. To evaluate the changes in pathogenicity of the A(H1N1)pdm09 viruses during their continued circulation in humans, we compared the nucleotide and amino acid sequences of ten A(H1N1)pdm09 viruses isolated in Japan between 2009 and 2015, and experimentally infected mice with each virus. The severity of infection caused by these Japanese isolates ranged from milder to more severe than that caused by the prototypic pandemic strain A/California/04/2009 (CA04/09); however, specific mutations responsible for their pathogenicity have not yet been identified.

## 1. Introduction

In the 20th and 21st centuries, human populations have experienced four influenza pandemics: the ‘Spanish influenza’ in 1918/1919, the ‘Asian influenza’ in 1957, the ‘Hong Kong influenza’ in 1968, and the ‘A(H1N1)pdm09’ virus, which emerged in Mexico in the spring of 2009 and rapidly spread worldwide within a few months [1,2,3]. This first influenza pandemic of the 21st century mainly caused mild symptoms in adults [4]; however, many cases of severe infection in healthy individuals without underlying health issues were also reported [5,6]. 

In fatal cases of human infection with A(H1N1)pdm09 virus during the pandemic, severe lung lesions, diffuse alveolar damage, and hemorrhagic interstitial pneumonitis were observed [7,8]. Pathological evaluation of those cases revealed A(H1N1)pdm09 virus antigen predominantly in the lung parenchyma [7,8,9], which is unusual in humans infected with seasonal influenza viruses, but often observed in fatal cases of human infection with highly pathogenic H5 avian influenza virus [10]. Experimental infection of mammalian models with A(H1N1)pdm09 viruses isolated early in the pandemic demonstrated that these viruses replicated more efficiently in the lungs and caused more severe viral pneumonia than contemporary circulating seasonal influenza viruses [11,12], suggesting that the initial isolates of the 2009 pandemic were more pathogenic in mammals than the pre-2009 pandemic seasonal influenza viruses. 

In the post-pandemic era, studies on the severity of A(H1N1)pdm09 virus infection have yielded inconsistent results, with one group reporting increased [13] severity of the virus and another reporting a decrease in severity [14]. It is unclear whether the changes in severity of the post-pandemic A(H1N1)pdm09 viruses are due to alterations in host or viral factors, such as herd immunity in humans, or are due to alterations in viral pathogenicity. To clarify this point, here we compared the nucleotide and amino acid sequences of A(H1N1)pdm09 viruses isolated during the pandemic and post-pandemic periods and evaluated the pathogenicity of these viruses in a mouse infection model.

## 2. Materials and Methods 

### 2.1. Cells and Viruses

Madin–Darby canine kidney (MDCK) cells were maintained in minimal essential medium (MEM) containing 5% newborn calf serum at 37 °C in 5% CO_2_. In the present study, we used the following ten A(H1N1)pdm09 viruses that were isolated between 2009 and 2015: A/Osaka/488/2009 (Osaka488/09), A/Osaka/83/2011 (Osaka83/11), A/Yokohama/UT-K101/2012 (YokohamaUTK101/12), A/Osaka/33/2013 (Osaka33/13), A/Osaka/UT-A01/2013 (OsakaUTA01/13), A/Osaka/6/2014 (Osaka6/14), A/Yokohama/50/2015 (Yokohama50/15), A/Yokohama/90/2015 (Yokohama90/15), A/Yokohama/94/2015 (Yokohama94/15), and A/Yokohama/100/2015 (Yokohama100/15). In addition to these Japanese isolates, A/California/04/2009 (CA04/09), the prototypic strain isolated early in the pandemic, was used as a reference strain. The accession numbers of the gene sequences of the viruses used in this study are shown in Table S1. The Japanese viruses were isolated from patients; unfortunately, detailed medical records, including vaccination history, treatment, and medical support after the onset of influenza in these patients were unavailable. CA04/09 was generated by reverse genetics as described elsewhere [15]. All Japanese strains were grown in MDCK cells to make stock viruses (no strain was passaged more than 4 times), and the viral genes of the stock viruses were sequenced.

### 2.2. Phylogenetic Analysis

The phylogenetic tree of A(H1N1)pdm09 virus was constructed using 124 whole amino acid sequences of hemagglutinin (HA) protein by the neighbor-joining method with Kimura distances and the 1000 replicates bootstrap using ClustalW 1.83 on the DDBJ (DNA Data Bank of Japan) website (http://clustalw.ddbj.nig.ac.jp/). The HA sequence of A/California/04/2009 was used as the outgroup to root the tree. The HA sequences of the A(H1N1)pdm09 isolates were obtained from the EpiFlu database (https://www.gisaid.org/), and the HA genetic clades were determined based on representative isolates of each clade available through the EpiFlu database.

### 2.3. Sequencing

Viral RNA was extracted from the culture supernatant of MDCK cells infected with each virus by using a QIAamp Viral RNA Mini Kit (QIAGEN, Hilden, Germany). The first-strand cDNA was synthesized by using Uni12 primer [16] and a SuperScript III (Invitrogen, Carlsbad, CA, USA). PCR was carried out with Phusion High-Fidelity DNA polymerase (NEB, Ipswich, MA, USA) and primer sets specific for A(H1N1) virus to amplify the viral genes of the Japanese isolates and the CA04/09 virus from the synthesized cDNA. The primer sequences used are shown in Table S2. The PCR products were purified with a gel extraction kit (QIAGEN, Hilden, Germany) and sequenced with a BigDye Terminator version 3.1 Cycle Sequencing Kit (Applied Biosystems, Foster City, USA) on an ABI PRISM 3130 DNA analyzer (Applied Biosystems, Foster City, USA).

### 2.4. Mouse Experiments

Six-week-old female BALB/c mice weighing 15 to 20 g (Japan SLC, Inc., Shizuoka, Japan) were used in this study. Baseline body weights were measured prior to infection. To determine the MLD_50_ values (mouse lethal dose 50; i.e., the dose required to kill 50% of infected mice), five mice per group were intranasally inoculated with 10^4^ to 10^6^ PFU (in 50 µl) of virus under sevoflurane anesthesia. Body weight and survival were monitored daily for 14 days post-infection. The percentage of body weight change was calculated by comparing the weight of each mouse at each timepoint to its initial weight on day 0. The percentage of maximum body weight loss was determined by comparing the greatest reduction in body weight for each mouse to its initial body weight. We euthanized mice when they developed >25% body weight loss and classified them as fatalities. The MLD_50_ values were calculated by using the method of Reed and Muench [17].

To assess virus growth in respiratory organs, nine mice per group were infected intranasally with 10^5^ PFU of virus; at days 3, 6, and 9 post-infection (dpi), three mice per group were euthanized and their lungs were collected, homogenized with MEM containing 0.3% bovine serum albmin, and titrated in MDCK cells by using plaque assays.

### 2.5. Statistical Analysis

Data were analyzed by using a one-way ANOVA and Dunnett tests for multiple comparisons; *p*-values of <0.05 were considered statistically significant.

Human H1N1 protein sequences were downloaded from the Influenza Research Database (www.fludb.org), on 12 October, 2019. Using IRD search filter options, we searched for human H1N1 protein sequences that were similar to pdmH1N1 2009, excluded laboratory strains, removed duplicated sequences, considered sequences from all geographic locations, and considered only isolates from the 2009 (April to September 2009) to the 2015–2016 seasons. In total, the numbers of sequences we analyzed were as follows: 1297 (polymerase basic 2; PB2), 1119 (polymerase basic 1; PB1), 1312 (polymerase acidic protein; PA), 4637 (HA), 2481 (neuraminidase; NA), 560 (nucleoprotein; NP), 374 (matrix 1 protein; M1), 356 (matrix 2 protein; M2), 777 (non-structural protein 1; NS1), and 284 (non-structural protein 2; NS2). Next, we aligned separately the sequences of each segment at each season, using MAFFT [18]. Finally, for each alignment, we used in-house scripts to determine the frequencies of each amino acid at each position of the alignments.

### 2.6. Ethics

A(H1N1)pdm09 viruses used in this study were isolated from patients by following a protocol approved by the Research Ethics Review Committee of the Institute of Medical Science, the University of Tokyo (approval numbers 30-77-B0304 and 26-42-0822; approved on March 4, 2018 and August 22, 2014, respectively). All experiments in this manuscript were performed in accordance with the University of Tokyo’s guidelines and regulations. Written informed consent was obtained from all participants.

All experiments with mice, including methods for sample collection and virological analysis, were performed in the biosafety level 2 containment laboratory in the Institute of Medical Science, the University of Tokyo (Tokyo, Japan) in accordance with the Regulations for Animal Care of the University of Tokyo and the Guidelines for Proper Conduct of Animal Experiments by the Science Council of Japan, and were approved by the Animal Experiment Committee of the Institute of Medical Science, the University of Tokyo (approval no. PA 15-10; approved on May 26, 2015).

## 3. Results

### 3.1. Genetic Analyses of Japanese A(H1N1)pdm09 Isolates

**Figure 1:**
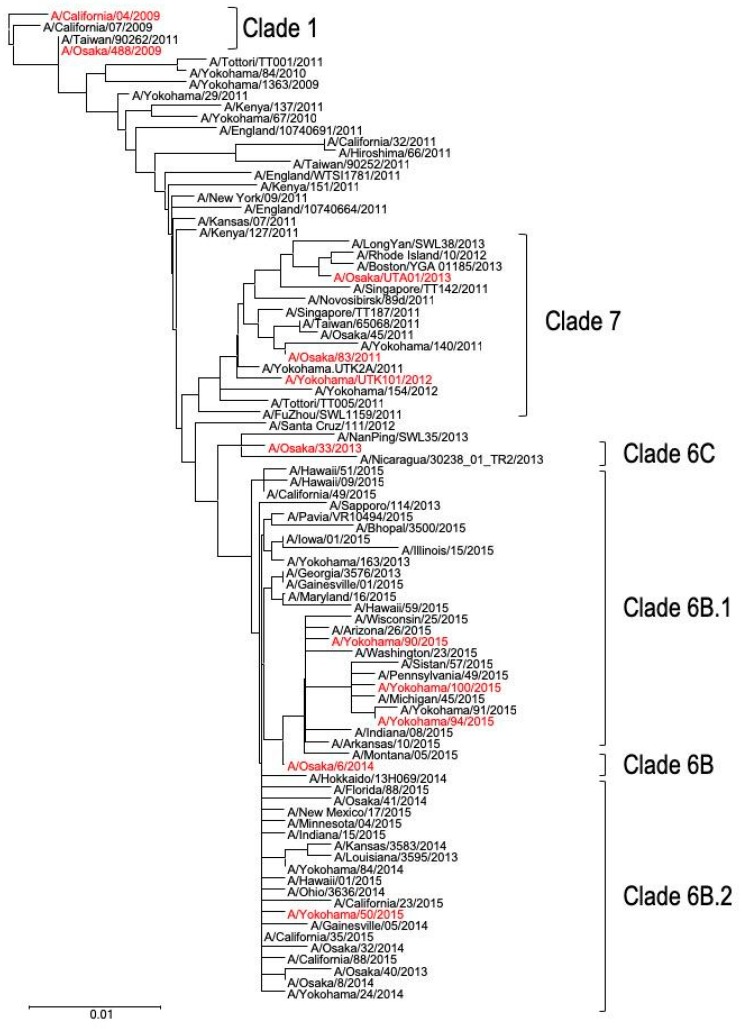
Phylogenetic tree based on hemagglutinin (HA) amino acid sequence. The trees were constructed by using the neighbor-joining method. Viral clades are indicated by brackets [19]. The Japanese isolates used in the present study are indicated in red.

To compare the pathogenicity of viruses isolated from patients during the pandemic with that of those isolated from patients in the post-pandemic era, we selected one Japanese isolate from the pandemic period and 1–3 isolates from each of the six clusters identified through phylogenetic analysis of their HA gene (Figure 1). A total of ten Japanese A(H1N1)pdm09 viruses isolated from 2009 up to the 2015–2016 season were analyzed: Osaka488/09, Osaka83/11, YokohamaUTK101/12, Osaka33/13, OsakaUTA01/13, Osaka6/14, Yokohama50/15, Yokohama90/15, Yokohama94/15, and Yokohama100/15. In addition to these Japanese isolates, CA04/09, the prototypic strain isolated early in the pandemic, was used as a reference strain.

Genetic analysis revealed that the nucleotide sequences of the eight segments of the Japanese isolates exhibited very high identities (>97.2%) with those of CA04/09 (Table 1). When we compared the amino acid sequences of the ten major viral proteins (HA, NA, PB2, PB1, PA, NP, M1, M2, NS1, and NS2) of the Japanese isolates with those of CA04/09, we found a total of 145 differences in the amino acid sequences of the Japanese isolates compared with those of CA04/09 (Table S3). These substitutions did not include virulence markers previously reported in A(H1N1)pdm09 viruses, such as the Asp-to-Gly change at position 222 of HA [20,21,22,23,24]. To evaluate whether these 145 amino acid substitutions are common among A(H1N1)pdm isolates, we examined the frequency with which they appear in reported A(H1N1)pdm isolate sequences in the Influenza Research Database (www.fludb.org) (Table S4). The numbers of strains per protein we analyzed were as follows: 1297 (PB2), 1119 (PB1), 1312 (PA), 4637 (HA), 2481 (NA), 560 (NP), 374 (M1), 356 (M2), 777 (NS1), and 284 (NS2). The frequency with which these substitutions have appeared in the viral proteins of the Japanese isolates has increased over time since 2009 (Table S4), suggesting that some of these mutations were gradually acquired, possibly to enhance viral fitness during circulation among humans.

### 3.2. Pathogenicity of A(H1N1)pdm09 Isolates in Mice

**Figure 2:**
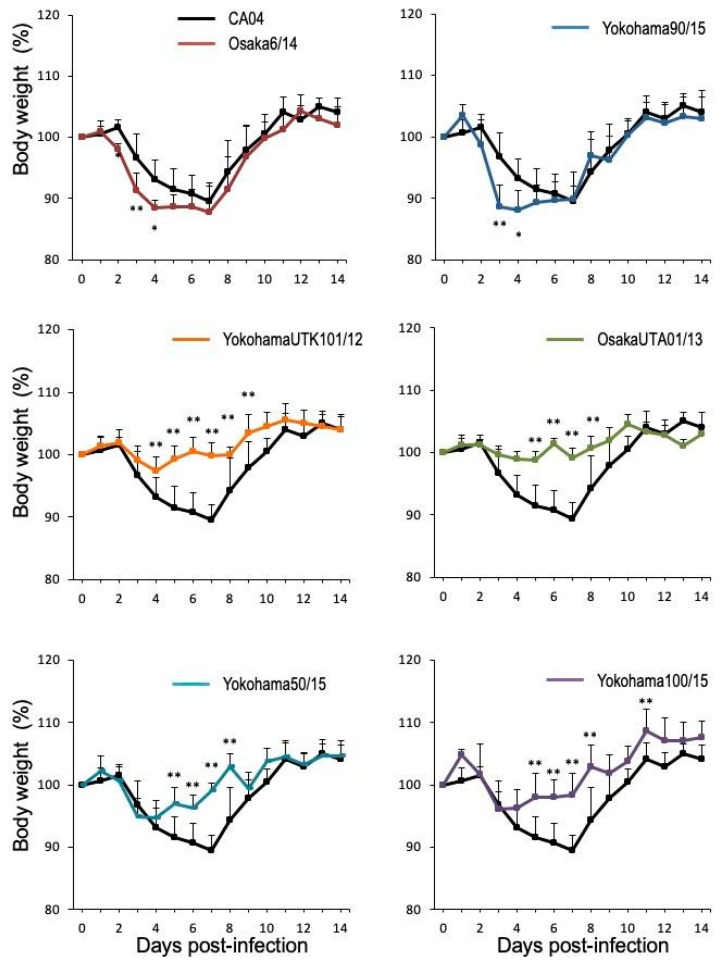
Body weight changes of mice infected with Japanese isolates or with CA04/09. Mice (five per group) were infected intranasally with 10^5^ PFU of each virus. Asterisks indicate the timepoints at which mice infected with Japanese isolates showed greater or smaller body weight loss than mice infected with CA04/09 (*, *p* < 0.05; **, *p <* 0.01).

To examine the pathogenicity of the Japanese A(H1N1)pdm09 isolates in vivo, five BALB/c mice per group were infected intranasally with 10^5^ PFU of each of the ten different Japanese viruses and CA04/09. The infected mice were monitored for body weight changes and mortality for up to 14 days (Figure S1). We also determined the MLD_50_. Of the Japanese isolates, only Osaka6/14 and Yokohama90/15 were lethal in infected mice (Table 2). Four of the ten isolates (i.e., Osaka488/09, Osaka83/11, Osaka33/13, and Yokohama94/15) caused similar mean maximum body weight loss to that caused by CA04/09 (Table 2), and the remaining four Japanese isolates (i.e., YokohamaUTK101/12, OsakaUTA01/13, Yokohama50/15, and Yokohama100/15) caused significantly less weight loss than did CA04/09 (Table 2 and Figure 2). In contrast, Osaka6/14 and Yokohama90/15, which were isolated in 2014 and 2015, respectively, exhibited relatively high pathogenicity in mice (Table 2, Figure 2 and Figure S1); their MLD_50_ values were lower than that of CA04/09 and they caused more severe body weight loss than CA04/09 did at 3 and 4 dpi (Figure 2).

We next examined the replicative ability of the Japanese isolates in mice. Mice were infected with 10^5^ PFU of the viruses and virus titers in the lungs were determined at 3, 6, and 9 dpi. All of the Japanese isolates replicated efficiently in the lungs of the infected mice at 3 and 6 dpi, whereas no virus was detected at 9 dpi (Table 3). Compared with CA04/09, YokohamaUTK101/12 and OsakaUTA01/13 replicated in mouse lung less efficiently, whereas Osaka6/14, Yokohama90/15, and Yokohama94/15 replicated more efficiently (Table 3). 

Taken together, these findings suggest that the pathogenicity and replicative ability of A(H1N1)pdm09 viruses isolated in Japan between 2009 and 2015 varies; however, the molecular basis for these differences in pathogenicity and replicative ability remain unknown.

## 4. Discussion

Here we demonstrated that the frequency with which specific substitutions arise in influenza viral proteins has increased from the pandemic to the post-pandemic era, and that the pathogenicity in mice of A(H1N1)pdm09 viruses isolated during and after the 2009 pandemic varies. Further studies will be required to determine which factors are associated with their pathogenicity and replicative ability. 

Previous studies demonstrated that viral replicative ability in the lungs reflects the virulence of the A(H1N1)pdm09 viruses [25,26,27]. Consistent with these previous observations, we found that the more pathogenic Japanese isolates (i.e., Osaka6/14 and Yokohama90/15) replicated more efficiently in mouse lung and less pathogenic Japanese isolates (i.e., YokohamaUT-K101/12 and OsakaUT-A01) replicated less efficiently in mouse lung compared with CA04/09. However, Yokohama50/15 and Yokohama100/15 were less pathogenic than CA04/09 despite having similar replicative ability to that of CA04/09, suggesting that factors other efficient replicative ability are important for the pathogenicity of A(H1N1)pdm09 viruses. Continuous monitoring of the virulence of A(H1N1)pdm09 viruses is essential for risk assessment of A(H1N1)pdm09 virus infection.

## Figures and Tables

**Table 1:** Nucleotide identities among the gene segments of the Japanese isolates and those of CA04/09 ^a^.

Viruses	Isolation Year	Nucleotide Identity with CA04/09 (%)							
		PB2	PB1	PA	HA	NP	NA	M	NS
Osaka488/09	2009	99.8	99.8	99.7	99.4	99.8	99.5	99.8	100.0
Osaka83/11	2011	99.1	99.4	99.1	98.8	99.4	99.1	99.2	99.0
YokohamaUTK101/12	2012	99.2	99.4	99.2	98.9	99.4	99.2	99.4	99.2
Osaka33/13	2013	98.6	98.9	98.6	98.4	98.7	98.9	**98.5**	97.8
OsakaUTA01/13	2013	98.8	99.1	98.6	98.3	98.8	98.9	**98.5**	98.4
Osaka6/14	2014	98.3	98.7	98.7	98.1	98.5	98.8	99.2	**97.2**
Yokohama50/15	2015	98.2	**98.2**	98.7	97.7	**98.0**	98.0	98.5	97.5
Yokohama90/15	2015	**98.1**	**98.2**	**98.1**	97.8	98.3	98.0	98.8	**97.2**
Yokohama94/15	2015	**98.1**	98.3	**98.1**	97.7	98.2	98.0	98.9	**97.2**
Yokohama100/15	2015	98.2	98.3	98.3	**97.5**	98.2	**97.8**	98.6	**97.2**

^a^ Nucleotide identities among the coding regions of the eight viral segments of the Japanese isolates and those of CA04/09 were determined. Segments of Osaka488/09 exhibited the highest nucleotide identities with those of CA04/09. The minimum nucleotide identities with CA04/09 are shown in boldface.

**Table 2:** Pathogenicity of the Japanese A(H1N1)pdm09 and CA04/09 viruses ^a^.

Virus	MLD_50_	% of Mean Maximum Body Weight Loss (mean ± SD) ^b^
CA04/09	>10^6.5^	10.5 ± 2.5
Osaka488/09	>10^6.5^	11.4 ± 4.2
Osaka83/11	>10^6.5^	9.1 ± 3.1
YokohamaUTK101/12	>10^6.5^	2.6 ± 2.3^**c^
Osaka33/13	>10^6.5^	7.6 ± 2.9
Osaka UTA01/13	>10^6.5^	1.2 ± 1.3^**^
Osaka 6/14	10^5.8^	12.3 ± 4.8
Yokohama50/15	>10^6.5^	5.4 ± 2.9^*^
Yokohama90/15	10^5.8^	11.8 ± 3.1
Yokohama94/15	>10^6.5^	8.6 ± 5.0
Yokohama100/15	>10^6.5^	3.9 ± 2.8^*^

^a^ BALB/c mice were infected intranasally with 10-fold serial dilutions of each virus (from 10^4^ to 10^6^ PFU per mice). Body weight and survival of infected mice were monitored daily for 14 days post-infection. ^b^ Maximum body weight loss of mice infected with 10^5^ PFU of virus. ^c^ Asterisks indicate that the body weight loss was significantly higher or lower in mice infected with the respective virus compared with that in mice infected with CA04/09 (*, *p* < 0.05; **, *p* < 0.01).

**Table 3:** Replicative ability of the Japanese A(H1N1)pdm09 and CA04/09 viruses ^a^.

Virus	Virus Titers (mean log_10_ PFU/g ±SD)in the Lung	
	Day 3	Day 6
CA04/09	7.2 ± 0.1	5.9 ± 0.7
Osaka488/09	7.2 ± 0.2	6.4 ± 0.1
Osaka83/11	7.1 ± 0.1	6.2 ± 0.2
YokohamaUT-K101/12	6.3 ± 0.2 ^** b^	5.3 ± 0.1
Osaka33/13	7.2 ± 0.2	6.1 ± 0.2
OsakaUT-A01/13	6.9 ± 0.1 ^*^	5.9 ± 0.3
Osaka6/14	7.8 ± 0.2 ^**^	6.2 ± 0.4
Yokohama50/15	7.4 ± 0.1	6.0 ± 0.8
Yokohama90/15	7.8 ± 0.2 ^**^	6.0 ± 0.2
Yokohama94/15	7.7 ± 0.1 ^**^	6.2 ± 0.2
Yokohama100/15	7.1 ± 0.1	5.9 ± 0.5

^a^ Mice (three mice per group) were infected with 10^5^ PFU of each virus and euthanized at 3, 6, and 9 days post-infection (dpi). Virus titers in the lung were determined by use of plaque assays. No virus was detected at 9 dpi. ^b^ Asterisks indicate that the virus titers were significantly higher or lower in the lungs of mice infected with that Japanese isolate compared with those in the lungs of mice infected with CA04/09 (*, *p* < 0.05; **, *p* < 0.01).

## Supplementary Materials

The following are available online at https://www.mdpi.com/1999-4915/12/2/155/s1, Figure S1: Survival of mice infected with viruses, Table S1: The accession numbers for the gene sequences of the viruses used in this study, Table S2: A list of primer sets specific for A(H1N1) virus used to amplify the viral genes of the Japanese isolates and the CA04/09 virus from the synthesized cDNA, Table S3: A list of primer sets specific for A(H1N1) virus used to amplify the viral genes of the Japanese isolates and the CA04/09 virus from the synthesized cDNA, Table S4: Frequency of amino acid substitutions at the respective positions of influenza A virus proteins.
