# Supplementary material for: Comparison of the Pathogenicity in Mice of A(H1N1)pdm09 Viruses Isolated between 2009 and 2015 in Japan
Source: Viruses. 2020 Jan 29;12(2):155. doi: 10.3390/v12020155 (PMC7077310; doi:10.3390/v12020155)
Supplement: Supplementary file 1 [file viruses-12-00155-s001.zip › Mitake_Viruses_Sup/Mitake_Viruses_rev_Sup Fig S1.pptx]

## Slide 1
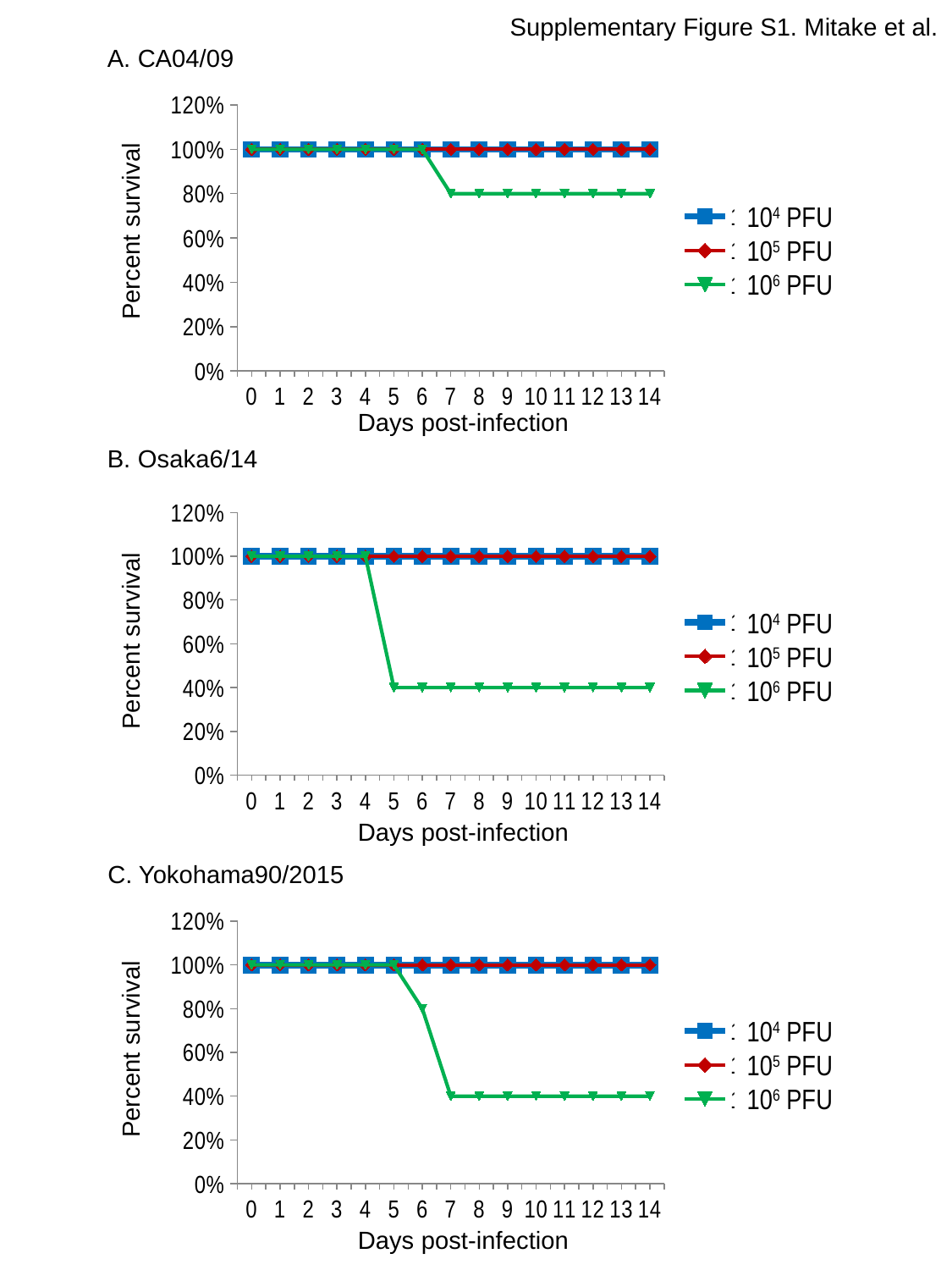

Supplementary Figure S1. Mitake et al.
A. CA04/09
### Chart
| Category | 10^4 | 10^5 | 10^6 |
|---|---|---|---|
| 0 | 1.0 | 1.0 | 1.0 |
| 1 | 1.0 | 1.0 | 1.0 |
| 2 | 1.0 | 1.0 | 1.0 |
| 3 | 1.0 | 1.0 | 1.0 |
| 4 | 1.0 | 1.0 | 1.0 |
| 5 | 1.0 | 1.0 | 1.0 |
| 6 | 1.0 | 1.0 | 1.0 |
| 7 | 1.0 | 1.0 | 0.8 |
| 8 | 1.0 | 1.0 | 0.8 |
| 9 | 1.0 | 1.0 | 0.8 |
| 10 | 1.0 | 1.0 | 0.8 |
| 11 | 1.0 | 1.0 | 0.8 |
| 12 | 1.0 | 1.0 | 0.8 |
| 13 | 1.0 | 1.0 | 0.8 |
| 14 | 1.0 | 1.0 | 0.8 |104 PFU
105 PFU
106 PFU
Percent survival
Days post-infection
B. Osaka6/14
### Chart
| Category | 10^4 | 10^5 | 10^6 |
|---|---|---|---|
| 0 | 1.0 | 1.0 | 1.0 |
| 1 | 1.0 | 1.0 | 1.0 |
| 2 | 1.0 | 1.0 | 1.0 |
| 3 | 1.0 | 1.0 | 1.0 |
| 4 | 1.0 | 1.0 | 1.0 |
| 5 | 1.0 | 1.0 | 0.4 |
| 6 | 1.0 | 1.0 | 0.4 |
| 7 | 1.0 | 1.0 | 0.4 |
| 8 | 1.0 | 1.0 | 0.4 |
| 9 | 1.0 | 1.0 | 0.4 |
| 10 | 1.0 | 1.0 | 0.4 |
| 11 | 1.0 | 1.0 | 0.4 |
| 12 | 1.0 | 1.0 | 0.4 |
| 13 | 1.0 | 1.0 | 0.4 |
| 14 | 1.0 | 1.0 | 0.4 |104 PFU
105 PFU
106 PFU
Percent survival
Days post-infection
C. Yokohama90/2015
### Chart
| Category | 10^4 | 10^5 | 10^6 |
|---|---|---|---|
| 0 | 1.0 | 1.0 | 1.0 |
| 1 | 1.0 | 1.0 | 1.0 |
| 2 | 1.0 | 1.0 | 1.0 |
| 3 | 1.0 | 1.0 | 1.0 |
| 4 | 1.0 | 1.0 | 1.0 |
| 5 | 1.0 | 1.0 | 1.0 |
| 6 | 1.0 | 1.0 | 0.8 |
| 7 | 1.0 | 1.0 | 0.4 |
| 8 | 1.0 | 1.0 | 0.4 |
| 9 | 1.0 | 1.0 | 0.4 |
| 10 | 1.0 | 1.0 | 0.4 |
| 11 | 1.0 | 1.0 | 0.4 |
| 12 | 1.0 | 1.0 | 0.4 |
| 13 | 1.0 | 1.0 | 0.4 |
| 14 | 1.0 | 1.0 | 0.4 |104 PFU
105 PFU
106 PFU
Percent survival
Days post-infection
