# Supplementary material for: Comparison of the Pathogenicity in Mice of A(H1N1)pdm09 Viruses Isolated between 2009 and 2015 in Japan
Source: Viruses. 2020 Jan 29;12(2):155. doi: 10.3390/v12020155 (PMC7077310; doi:10.3390/v12020155)
Supplement: Supplementary file 1 [file viruses-12-00155-s001.zip › Mitake_Viruses_Sup/Mitake_Viruses_rev_Sup Info.docx]

**Supplementary Information**

- Supplementary Table S1. The accession numbers for the gene sequences of the viruses used in this study
- Supplementary Table S2. A list of primer sets specific for A(H1N1) virus used to amplify the viral genes of the Japanese isolates and the CA04/09 virus from the synthesized cDNA
- Supplementary Table S3 (Excel sheet)
- Supplementary Figure legend

**Supplementary Table S1. The accession numbers for the gene sequences of the viruses used in this study.**

**Supplementary Table S2. A list of primer sets specific for A(H1N1) virus used to amplify the viral genes of the Japanese isolates and the CA04/09 virus.**

| Primer | Sequence (5' - 3') | Orientation | Purpose |
| --- | --- | --- | --- |
| PB2 U12+18F | AGCGAAAGCAGGTCAAATATATTCAATATG | Forward | Amplification of PB2 fragment |
| PB2 U13+18R | AGTAGAAACAAGGTCGTTTTTAAACAATTCG | Reverse | Amplification of PB2 fragment |
| PB1 U12+15F | AGCAAAAGCAGGCAAACCATTTGAATG | Forward | Amplification of PB1 fragment |
| PB1 U13+16R | AGTAGAAACAAGGCATTTTTTCATGAAGG | Reverse | Amplification of PB1 fragment |
| PA U12+15F | AGCAAAAGCAGGTACTGATCCAAAATG | Forward | Amplification of PA fragment |
| PA U13+20R | AGTAGAAACAAGGTACTTTTTCGGACAGTATGG | Reverse | Amplification of PA fragment |
| HA U12+16F | AGCAAAAGCAGGGGAAAATAAAAGCAAC | Forward | Amplification of HA fragment |
| HA U13+16R | AGTAGAAACAAAGGGTGTTTTTTCTCATG | Reverse | Amplification of HA fragment |
| NP U12+18F | AGCAAAAGCAGGGTAGATAATCACTCAATG | Forward | Amplification of NP fragment |
| NP U13+13R | AGTAGAAACAAGGGTATTTTTCCTCA | Reverse | Amplification of NP fragment |
| NA U12+15F | AGCAAAAGCAGGAGTTCAAAATGAATC | Forward | Amplification of NA fragment |
| NA U13+17R | TTATATGGTCTCGTATTAGTAGAAACAAGG | Reverse | Amplification of NA fragment |
| M U12+16F | AGCAAAAGCAGGTAGATATTTAAAGATG | Forward | Amplification of M fragment |
| M U13+7R | AGTAGAAACAAGGTAGTTTT | Reverse | Amplification of M fragment |
| NS U12+14F | AGCAAAAGCAGGGTGACAAAGACATA | Forward | Amplification of NS fragment |
| NS U13+17R | AGTAGAAACAAGGGTGTTTTTTATCA | Reverse | Amplification of NS fragment |

**Supplementary Figure legend**

**Supplementary Figure S1. Survival of mice infected with viruses.**

Mice (five per group) were infected intranasally with 10^4^, 10^5^, or 10^6^ PFU of (A) CA04/09, (B) Osaka6/14, or (C) Yokohama90/15. Survival was monitored daily for 14 days.
